# Association between Normal Thyroid Hormones and Diabetic Retinopathy in Patients with Type 2 Diabetes

**DOI:** 10.1155/2020/8161797

**Published:** 2020-02-12

**Authors:** Jian Zou, Zeping Li, Feng Tian, Yi Zhang, Chao Xu, Jiajia Zhai, Min Shi, Guangxian Wu, Zheng Zhang, Chao Yang, Haixu Chen, Xiaomiao Li

**Affiliations:** ^1^Department of Medicine, The 522 Hospital of the Chinese PLA, Luoyang 471003, China; ^2^Department of Endocrinology and Metabolism, The First Affiliated Hospital of Air Force Military Medical University (Fourth Military Medical University), Xi'an 710032, China; ^3^Queen Marry College, Nanchang University, Nanchang 330000, China; ^4^Department of Endocrinology and Metabolism, Xi'an Ninth Hospital, Xi'an 710054, China; ^5^Medical Department, Luoyang Fifth People's Hospital, Luoyang 471003, China; ^6^PLA Rocket Force Characteristic Medical Center, Beijing 100088, China; ^7^Institute of Geriatrics & National Clinical Research Center of Geriatrics Disease, Chinese PLA General Hospital, Beijing 100853, China

## Abstract

The relationship between normal thyroid function and type 2 diabetes mellitus (T2DM) has been a particular focus for concern. The present study determined the relationship between thyroid hormone levels and the prevalence of diabetic retinopathy (DR) in T2DM patients. A cross-sectional study (*n* = 633) was performed in Xi'an, Shaanxi Province, China. Subjects were evaluated for anthropometric measurements, thyroid function, and diabetic retinopathy. Logistic regression models were used to assess the relationships between thyroid hormones and DR. Of 633 patients, 243 (38.4%) patients suffered from DR. The prevalence of DR showed a significantly decreasing trend across the quartiles based on free triiodothyronine (FT3) (FT3 quartile 1 group [FT3-Q1] <4.35 pmol/L, FT3 quartile 2 group [FT3-Q2] 4.35–4.70 pmol/L, FT3 quartile 3 group [FT3-Q3] 4.70–5.08 pmol/L, and FT3 quartile 4 group [FT3-Q4] ≥5.08 pmol/L) (56.7%, 42.5%, 33.1%, 23.8%, *P* < 0.001). In comparison with all participants categorized in FT3-Q1, the multivariable adjusted odds ratios (95% confidence interval) of DR in FT3-Q2, FT3-Q3, and FT3-Q4 were 0.587 (0.340–1.012), 0.458 (0.258–0.813), and 0.368 (0.201–0.673), (*P* = 0.055, *P* = 0.008, *P* = 0.001), respectively. FT3 levels within the normal range are negatively associated with DR in euthyroid patients with type 2 diabetes. Further studies should be aimed at clarifying the relationship between thyroid hormones and T2DM.

## 1. Introduction

The prevalence of type 2 diabetes mellitus (T2DM) is rising worldwide. Diabetic retinopathy (DR) is one of the most common microvascular complications of T2DM, leading to an increase in the socioeconomic burden [1]. According to the World Health Organization (WHO), the blindness caused by DR accounted for 4.8% of the total 37 million cases of blindness around the world in 2006 [2].

Numerous epidemiological studies [3–5] have indicated that diabetes mellitus (DM) patients are more susceptible to thyroid dysfunction than the general population is. The relationship between thyroid function and T2DM has been a particular focus for concern, and this relationship has extended to the field of euthyroidism. One study [6] addressing the relationship between thyroid function and the prevalence of T2DM was performed among 15,269 Chinese T2DM patients with normal thyroid function. In this cohort study, the prevalence of T2DM was negatively correlated with normal free triiodothyronine (FT3) and positively correlated with normal free thyroxine (FT4) in T2DM patients. Furthermore, there is a distinct lack of relevant research on the relationship between microvascular complications in T2DM and normal thyroid hormones. Qi et al. [7] demonstrated no association between high thyroid stimulating hormone (TSH) levels and an increased risk of DR. To date, only one study conducted by Wu et al. [8] attempted to elucidate these issues; the results showed that high normal levels of FT3 were negatively correlated with the incidence of diabetic kidney disease (DKD), but FT3 levels were not associated with DR in T2DM patients with normal thyroid function. We have previously deeply studied the relationship between normal thyroid function and DKD. In addition, our data showed that FT3 in normal range was negatively correlated with DKD in patients with T2DM [9]. Therefore, we conducted this study in the northwestern region of China to examine the relationship between normal thyroid hormone levels and DR.

## 2. Methods

### 2.1. Patients

We set up a database of T2DM inpatients at the First Affiliated Hospital of Air Force Military Medical University (Fourth Military Medical University). Some data in the database, which was concerning DKD and normal thyroid hormone [9], has been published. This present cross-sectional study analyzed data from the database from 2014 to 2016. According to the American Diabetic Association (ADA) 2013 criteria [10], T2DM was assessed as having a history of T2DM, or fasting plasma glucose (FPG) level ≥126 mg/dL (7.0 mmol/L), or oral glucose tolerance test ≥200 mg/dL (11.1 mmol/L), or HbA1c ≥6.5%. The following participants were excluded from the study: with acute complications of T2DM, with cataract, with thyroid disease in history, with taking drugs affecting glucose metabolism and thyroid function, and with malignant tumors. Pregnant or lactating women were also excluded. Consequently, there were 633 adult subjects with normal thyroid function in all recruited and all the subjects were the Han nationality. Informed consent was received from all subjects. The study followed the Declaration of Helsinki guidelines. The Ethics Committee at the First Affiliated Hospital of Air Force Military Medical University (Fourth Military Medical University) approved the study.

### 2.2. Data Collection

Demographic and anthropometric parameters, including age, gender, diabetes duration, the status of drinking and smoking, hypertension history, and medical history, were collected from the medical records. Blood pressure was measured in the sitting position after a rest period of more than 10 minutes. Patients having systolic blood pressure (SBP) ≥140 mmHg or diastolic blood pressure (DBP) ≥90 mmHg or having a history of hypertension were considered hypertensive. Body mass index (BMI) was calculated as weight in kilograms (kg) divided by the square of height in meters (m^2^), and waist-to-height ratio (WHtR) was calculated as waist divided by height in meters. We also obtained results of the following variables from measurement: FPG, HbA1c, thyroid function, serum low-density lipoprotein cholesterol (LDL-C), serum high-density lipoprotein cholesterol (HDL-C), serum total cholesterol (TC), and serum triglyceride (TG). Serum TSH, FT3, FT4, and thyroid peroxidase antibodies (TPO-Ab) were measured using chemiluminescence immunoassay (ADVIA Centaur Siemens New York, USA) and their normal references were defined as 0.35–5.5 *μ*IU/ml, 3.5–6.5 pmol/L, 11.5–22.7 pmol/L, and <78 U/mL respectively. As with the methods used in previous publication [9], all subjects were classified into 2 groups based on the level of TSH (<2.5 *μ*IU/mL and ≥2.5 *μ*IU/mL) and TPO-Ab (negative TPO-Ab and positive TPO-Ab), and 4 groups (quartiles) based on the level of FT3 (<4.35, 4.35–4.70, 4.70–5.08, and ≥5.08 pmol/L) and FT4 (<14.98, 14.98–16.23, 16.23–17.79, and ≥17.79 pmol/L), respectively.

### 2.3. Assessment of Diabetic Retinopathy

All subjects were evaluated by two qualified retinal photography using a TRC-6S (Topcon, Tokyo, Japan) nonmydriatic camera at 45° (two eyes × two fields). According to the international clinical DR severity scale [11], the severity of DR was graded as follows: (1) normal, (2) mild and moderate nonproliferative diabetic retinopathy (NPDR), (3) severe NPDR, and (4) proliferative diabetic retinopathy (PDR).

### 2.4. Statistical Analysis

Continuous variables were expressed as mean ± standard deviation, variables with a skewed distribution were expressed as an interquartile range, and categorical variables were expressed as percentages. The characteristics of the participants between the DR group and the non-DR group were compared using Chi-square tests, or Mann–Whitney *U* test, unpaired Student's *t*-tests, as appropriate. FT3 levels in different stages of DR were determined by analysis of variance (ANOVA) test. Logistic regression analyses were performed to estimate the risk of DR in different TSH, FT3, FT4, and TPO-Ab groups. We analyzed the unadjusted model and the adjusted model for the covariates. A *P* value <0.05 at the two-tailed level was considered statistically significant. All statistical analyses were performed using SPSS software version 16.0.

## 3. Results

### 3.1. Clinical Characteristics of the Participants

Table 1 lists the demographic, clinical, and biochemical data of the study participants. No significant differences between the DR and the non-DR groups were found in the treatment rate of metformin, BMI, WHtR, DBP, HbA1c, FPG, LDL-C, HDL-C, TG, and TC. The DR group had a higher proportion of older and female participants and those using insulin, a higher prevalence of hypertension, and longer duration of diabetes than the non-DR group did. In addition, the patients with DR showed higher SBP and lower hemoglobin (HGB) than those without DR (*P* < 0.05). The ratios of smoking and drinking between the two groups were also significantly different (*P* < 0.05). Moreover, the patients with DR also experienced lower FT3 than those in the non-DR group (*P* < 0.05), whereas the level of TSH and FT4 and the rate of positive TPO-Ab have no significant difference between the two groups.

### 3.2. The Prevalence of DR among FT3, FT4, TSH, and TPO-Ab Groups

**Figure 1:**
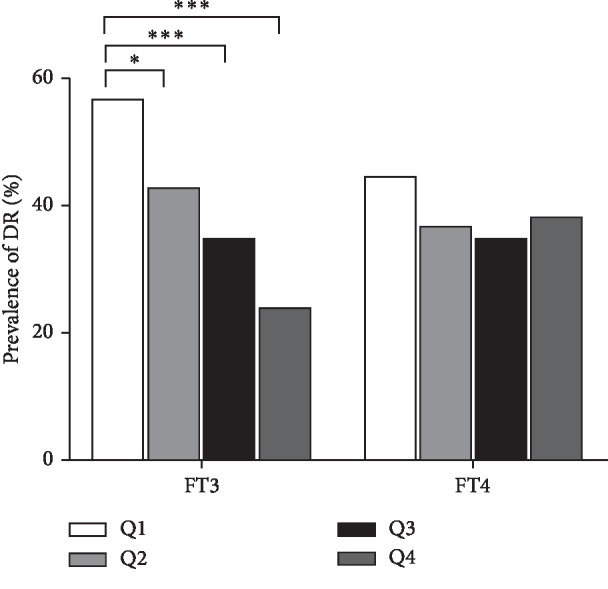
Prevalence of DR in different FT3 or FT4 quartiles (^*∗*^*P* < 0.05, ^*∗∗∗*^*P* < 0.001); FT3-Q1:FT3 quartile 1 (FT3 <4.35 pmol/L), FT3-Q2:FT3 quartile 2 (4.35 pmol/L ≤ FT3 <4.70 pmol/L), FT3-Q3:FT3 quartile 3 (4.70 pmol/L ≤ FT3 <5.08 pmol/L), FT3-Q4:FT3 quartile 4 (FT3 ≥5.08 pmol/L). FT4-Q1:FT4 quartile 1 (FT4 <14.98 pmol/L), FT4-Q2:FT4 quartile 2 (14.98 pmol/L ≤ FT4 <16.23 pmol/L), FT4-Q3:FT4 quartile 3 (16.23 pmol/L ≤ FT4 <17.79 pmol/L), FT4-Q4:FT4 quartile 4 (FT3 ≥17.79 pmol/L) (*n* = 633).

**Figure 2:**
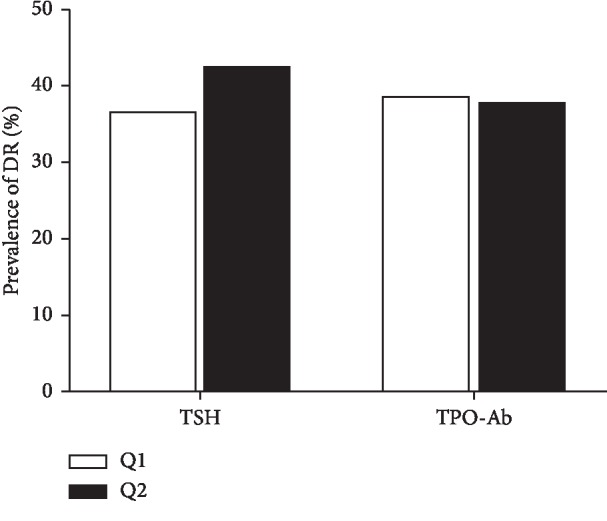
Prevalence of DR in different levels of TSH and TPO-Ab groups. TSH 1: <2.5 *μ*IU/mL, TSH 2 ≥ 2.5 *μ*IU/mL, TPO-Ab 1 < 78 U/mL, TPO-Ab 2 ≥ 78 U/ml (*n* = 633).

All subjects were classified into 2 groups based on the level of TSH (<2.5 *μ*IU/mL and ≥2.5 *μ*IU/mL) and TPO-Ab (negative TPO-Ab and positive TPO-Ab), and 4 groups (quartiles) based on the level of FT3 (<4.35, 4.35–4.70, 4.70–5.08, and ≥5.08 pmol/L) and FT4 (<14.98, 14.98–16.23, 16.23–17.79, and ≥17.79 pmol/L), respectively. According to the quartiles based on FT3 levels, the prevalence of DR exhibited a significantly declining tendency (56.7%, 42.5%, 33.1%, 23.8%, *P* < 0.001). Furthermore, compared with FT3 quartile 1 group (FT3-Q1), patients in FT3 quartile 2 group (FT3-Q2), FT3 quartile 3 group (FT3-Q3), and FT3 quartile 4 group (FT4-Q4) had a higher risk of DR (*P*=0.016, *P* < 0.001, *P* < 0.001), while the differences among FT4 groups, TSH groups, and TPO-Ab groups in the prevalence of DR was not statistically significant (Figures 1 and 2).

We stratified 4 groups based on the stages of DR. One-way analysis of variance was performed for the difference of FT3 levels among the 4 groups. The mean values of FT3 were significantly different according to the different stages of diabetic retinopathy (*P* < 0.001). When the differences of FT3 levels among several groups were statistically significant, the multiple comparisons were carried out. Compared to the normal group, the other 3 groups had a higher FT3, respectively, by LSD *t*-test (*P*=0.002, *P* < 0.001, *P* < 0.001). (Table 2).

### 3.3. Relationship of Thyroid Hormone Levels and TPO-Ab with the Prevalence of DR

Table 3 presents the results of logistic regression analysis to identify FT3, FT4, TSH, and TPO-Ab associated with the prevalence of DR. Compared to those in FT3-Q1 (Q1 <4.35), the crude ORs (95% CI) for DR in FT3-Q2 (4.35 ≤Q2 <4.70), FT3-Q3 (4.70 ≤Q3 <5.08), and FT3-Q4 (Q4 ≥5.08) were 0.563 (0.358–0.885), 0.378 (0.235–0.608), and 0.238 (0.144–0.392) (*P*=0.013, *P* < 0.001, *P* < 0.001) (Model 1), respectively. When adjusted for age, duration of T2DM, gender, smoking rate, BMI, the usage of insulin and metformin, hypertension, HbA1c, TG, and TC (Model2), the ORs (95% CI) for DR in FT3-Q2, FT3-Q3, and FT3-Q4 were 0.587 (0.340–1.012), 0.458 (0.258–0.813), and 0.368 (0.201–0.673) (*P*=0.055, *P*=0.008, *P*=0.001, respectively). Yet no correlation with DR in TSH, FT4, and TPO-Ab groups was found. The results also suggested that a significant independent negative association between FT3 and DR was found.

## 4. Discussion

In the cross-sectional study, 633 T2DM patients were enrolled. The prevalence of DR was 38.4%, which was comparable to that reported in the Journal of Clinical Diagnosis and Treatment of Diabetic Retinopathy (2014 version) [12]. On the basis of the results of an epidemiological study, which found that more than 95% of the normal population has TSH levels <2.5 *μ*IU/mL, so the upper limit of TSH recommended by the National Academy of Clinical Biochemistry (NACB) was 2.5 *μ*IU/mL [13]. Consequently, participants were categorized as the TSH <2.5 group and TSH ≥2.5 group, negative TPO-Ab and positive TPO-Ab groups, and FT3 and FT4 quartile groups in the present study, respectively. Our data suggested that the patients with DR had a lower FT3 level than those with non-DR (*P* < 0.05). Moreover, the prevalence of DR showed a declining trend with the increase of FT3 levels (*P* < 0.001). FT3 was independently associated with the prevalence of DR in logistic regression after adjustment, while no significant difference was noted with TSH, FT4, and TPO-Ab groups and DR.

So far, the association between normal thyroid hormone levels and DR with T2DM patients has not been studied thoroughly. In a recently published study [6] on Chinese populations, it was confirmed that the prevalence of T2DM was negatively correlated with FT3 and positively correlated with FT4, and this correlation was primarily determined by the level of FT3. It reported that there is no correlation between TSH and DR [7], but the values of FT3 and FT4 were not provided in this study. Moreover, another study from China [8] found that high normal levels of FT3 and DKD were negatively correlated but failed to find the relationship between DR and FT3 in euthyroid patients. The reasons that our findings were not consistent with that of the foregoing study [8] may be as follows: First, the prevalence of DR in the present study was 38.4%, compared with 29.9% in the previous study. Second, the level of FT3 in the present study was also higher than this study (4.72 ± 0.51 vs. 3.88 ± 0.42), although the reference ranges of FT3 in the two laboratories were slightly different (3.5–6.5 pmol/L vs. 3.1–6.8 pmol/L). In addition, the difference in FT3 levels may be the vital reason for the discrepancy between the result of our study and the previous findings.

None of the ORs was statistically significant among the TSH groups. It was consistent with the study by Wu et al. [8] and the study by Qi et al. [7], but the results differed from those reported by Yang et al. [14], which drew the conclusions that a subgroup with a higher TSH level (2.0 ≤ TSH < 4.0 IU/ml) had a significantly higher rate of sight-threatening diabetic retinopathy (STDR), compared to a lower TSH (0.4 ≤ TSH < 2.0 IU/ml) level group. All three of the above studies were conducted in the Asian population. In contrast, a study [15] in the Caucasian population found no correlation between TSH and DR in T2DM patients. It is important to note that in the abovementioned studies, only Wu et al. [8] enrolled subjects with normal thyroid function in T2DM patients, and three other studies [7, 14, 15] enrolled individuals with normal thyroid function and subclinical hypothyroidism (SCH) in T2DM patients. Unfortunately, these studies [7, 14, 15] only assessed the level of TSH, and FT3 was not measured. What is interesting, the study conducted on T1DM patients [16] showed that the higher FT3 concentration was related to the lower prevalence of microvascular complications and better metabolic control of the disease in adult euthyroid people.

The relationship between normal thyroid hormone levels and DR in T2DM patients is unclear, yet multiple mechanisms are possibly related to this relationship. Thyroid function is primarily responsible for the regulation of energy balance and metabolism [17]. Thyroid hormones can cause an increase of 6-phosphoglucose and glucose transporter 2 in liver cells and hepatic glucose output and thus promote hepatic gluconeogenesis. In addition, thyroid dysfunction decreases glucose transport in myocytes and increases insulin resistance in muscle and adipose tissue [18, 19]. Moreover, recent studies have demonstrated that FT3 can regulate insulin secretion [20, 21]. Therefore, FT3 may play an important role in glucose metabolism and insulin secretion. In addition, T3 was verified as affecting endothelial function by inducing relaxation of vascular smooth muscle in the experimental models [22, 23]. The study reported by Cai et al. [24] also found that T3 can play a role in relieving diabetic endothelial dysfunction in the arteries of diabetic rat, whereas inflammatory response and endothelial dysfunction play an important role in the development of DR. Therefore, FT3 levels may be closely related to DR through endothelial function.

However, our study did not find a correlation between FT4 and DR, as in the preceding study's result [8]. In agreement with this study [8], we also failed to find a relationship among TSH, TPO-Ab, and DR. The current results were in agreement with those reported by Qi et al. [7], which demonstrated no association between TSH and DR. Reasons may be that, first, the roles of thyroid hormones mainly act through FT3 binding to receptors to regulate transcription of target genes and expression of protein. Second, TPO-Ab-positive patients were not excluded. In general, TSH levels in TPO-Ab-positive individuals tended to be higher than in TPO-Ab-negative individuals, even in patients with normal thyroid function [25]. Furthermore, the low TPO-Ab-positive rate in the study may also contribute to this result (9.1%). Last, it was reported that metformin may lower TSH levels in T2DM patients [26]. Although we adjusted for the factor of metformin usage in the multiple logistic regression model, this factor may have some impact on the results.

Several limitations in the present study need to be explained. First, it is impossible to infer causality because of the cross-sectional design of this study. Thus, further studies are warranted to establish the relative risk between TH and DR in T2DM patients. Second, due to the hospitalization time limit, the thyroid function of subjects was assayed only once, so there may be some statistical bias. Third, diabetes is more likely to develop the low-T3 syndrome, which is prone to hypothyroidism [27, 28], whereas the reverse triiodothyronine (rT3) of the subjects was not assessed in the present study, so the association between thyroid hormone and DR may have been underestimated in our study. Fourth, according to the ADA 2013 criteria [10], high-quality fundus photographs can detect most clinically significant diabetic retinopathy. However, 45° fundus photography could be missing quite a bit of peripheral DR. Therefore, the prevalence of DR may be underestimated. Fifth, the present study did not assess urinary iodine concentration (UIC). China has been assessed as a country of more than adequate iodine intake by the World Health Organization (WHO) [29]. A cross-sectional study conducted in China demonstrated that the prevalence of clinical hypothyroidism, subclinical hypothyroidism, and positive thyroid antibodies was significantly higher in more than adequate iodine intake (MTAII) cities than inadequate iodine intake (AII) cities [30]. Although the subjects we enrolled were T2DM patients with normal thyroid function, the TSH level in the study participants may be affected by iodine intake, which may influence the study results. Lastly, all the subjects were from a single center.

From the results of the present study, we suggested that decreased FT3 was significantly related to the prevalence of DR in T2DM with normal thyroid function. These findings may provide the basis for additional large-scale cohort research. Prospective cohort studies are also warranted to assess the relation between thyroid hormones and DR in T2DM patients.

## Figures and Tables

**Table 1:** Clinical characteristics of T2DM patients with or without DR.

Characteristics	Total (*n* = 633)	Non-DR (*n* = 390)	DR (*n* = 243)	*P*
Age (*y*)	52.60 ± 10.59	49.59 ± 10.30	57.44 ± 9.18	<0.001
Gender (male), *n* (%)	444 (70.1)	298 (76.4)	146 (60.1)	<0.001
Duration of T2DM (years)	8.43 ± 6.33	5.93 ± 5.28	12.45 ± 5.80	<0.001
Smoking, *n* (%)				<0.001
No smoking	340 (53.7)	183 (46.9)	157 (64.6)	
Smoking	242 (38.2)	178 (45.6)	64 (26.3)	
Quit smoking	51 (8.1)	29 (7.4)	22 (9.1)	
Drinking, *n* (%)				<0.001
No drinking	453 (71.6)	251 (64.4)	202 (83.1)	
Drinking	157 (24.8)	123 (31.5)	34 (14.0)	
Quit drinking	23 (3.6)	16 (4.1)	7 (2.9)	
Hypertension, *n* (%)	254 (40.1)	125 (32.1)	129 (53.1)	<0.001
Insulin, *n* (%)	393 (62.1)	220 (56.4)	173 (71.2)	<0.001
Metformin, *n* (%)	401 (63.3)	257 (65.9)	144 (59.3)	0.107
BMI (kg/m^2^)	25.85 ± 3.36	25.95 ± 3.36	25.70 ± 3.37	0.368
WHtR	0.92 ± 0.06	0.92 ± 0.06	0.93 ± 0.07	0.742
SBP (mmHg)	128.62 ± 17.38	125.48 ± 16.68	133.67 ± 17.33	<0.001
DBP (mmHg)	80.08 ± 10.73	79.47 ± 10.96	81.05 ± 10.28	0.071
HbA1c (%)	8.86 ± 2.01	8.81 ± 2.05	8.94 ± 1.95	0.412
FPG (mmol/L)	8.88 ± 3.19	8.77 ± 3.22	9.04 ± 3.12	0.296
TC (mmol/L)	4.14 ± 1.08	4.10 ± 0.95	4.22 ± 1.26	0.179
TG (mmol/L)	1.57 (1.07, 2.38)	1.59 (1.11, 2.47)	1.45 (1.00, 2.27)	0.160
LDL-C (mmol/L)	2.38 ± 0.79	2.35 ± 0.76	2.43 ± 0.83	0.197
HDL-C (mmol/L)	0.98 ± 0.25	0.97 ± 0.24	1.00 ± 0.26	0.133
HGB (g/L)	141.69 ± 15.47	145.07 ± 13.31	136.27 ± 17.09	<0.001
TSH (uIU/ml)	2.01 (1.37, 2.89)	1.95 (1.37, 2.81)	2.08 (1.36, 2.95)	0.235
FT3 (pmol/L)	4.72 ± 0.51	4.83 ± 0.50	4.56 ± 0.47	<0.001
FT4 (pmol/L)	16.42 ± 2.13	16.46 ± 2.02	16.36 ± 2.30	0.573
Positive TPO-Ab, *n* (%)	40 (6.3)	25 (6.4)	15 (6.2)	1.000

Values are expressed as means ± SD or median (range) or count and percentage. BMI: body mass index; DR: diabetic retinopathy; DBP: diastolic blood pressure; FPG: fasting plasma glucose; FT3: free triiodothyronine; FT4: free thyroxine; HDL-C: high-density lipoprotein cholesterol; HGB: hemoglobin; LDL-C: low-density lipoprotein cholesterol; SBP: systolic blood pressure; TC: total cholesterol; TG: triglycerides; TPO-Ab: thyroid peroxidase antibody; TSH: thyroid stimulating hormone; WHtR: waist-to-height ratio.

**Table 2:** FT3 levels based on different stages of DR.

Group	Number	FT3 (pmol/L)	*P*
Normal	390	4.83 ± 0.50	
Mild and moderate NPDR	154	4.69 ± 0.46	0.002^a^
Severe NPDR	47	4.27 ± 0.34	<0.001^a^
PDR	42	4.40 ± 0.49	<0/001^a^

Data are shown as mean ± SD. *P* < 0.001 for difference among groups by analysis of variance (ANOVA) test and LSD *t*-test for multiple comparisons. ^a^:compared with the Normal group. NPDR: nonproliferative diabetic retinopathy; PDR: proliferative diabetic retinopathy.

**Table 3:** Logistic regression analysis of thyroid hormone levels with DR.

Characteristics	Groups	Number	Model 1 OR (95% CI)	*P*	Model 2 OR (95% CI)	*P*
TSH	<2.5	413	Reference		Reference	
	≥2.50	220	1.284 (0.919–1.794)	0.143	0.917 (0.605–1.390)	0.682
FT3	Q1 (<4.35)	154	Reference		Reference	
	Q2 (4.35–4.70)	159	0.563 (0.358–0.885)	0.013	0.587 (0.340–1.012)	0.055
	Q3 (4.70–5.08)	160	0.378 (0.235–0.608)	<0.001	0.458 (0.258–0.813)	0.008
	Q4 (≥5.08)	160	0.238 (0.144–0.392)	<0.001	0.368 (0.201–0.673)	0.001
FT4	Q1 (<14.98)	157	Reference		Reference	
	Q2 (14.98–16.23)	159	0.714 (0.455–1.120)	0.143	0.712 (0.411–1.235)	0.227
	Q3 (16.23–17.79)	159	0.657 (0.418–1.035)	0.070	0.738 (0.419–1.300)	0.293
	Q4 (≥17.79)	158	0.761 (0.485–1.193)	0.234	1.166 (0.666–2.041)	0.591
TPO-Ab	<78	593	Reference		Reference	
	≥78	40	0.961 (0.496–1.861)	0.905	0.979 (0.452–2.121)	0.958

Model 1: crude model. Model 2: adjusted for age, duration of T2DM, gender, smoking, BMI, insulin medication, metformin medication, hypertension, HbA1c, TG, and TC.

## Data Availability

The data used to support the findings of this study are available from the corresponding author upon request.
